# Preliminary outcomes following revision total knee arthroplasty using a new fixed-bearing revision knee system in Asians: a mean of 3-year follow-up

**DOI:** 10.1186/s13018-023-03503-3

**Published:** 2023-01-06

**Authors:** Oog-Jin Shon, Gi Beom Kim, Hyuck Goo Kim

**Affiliations:** 1 Present Address: Department of Orthopedic Surgery, Yeungnam University College of Medicine, 170 Hyeonchung‑ro Nam‑gu, Daegu, 42415 Republic of Korea; 2 Department of Anesthesia and Pain Medicine, Yeungnam University College of Medicine, 170 Hyeonchung‑ro Nam‑gu, Daegu, 42415 Republic of Korea; 3 Department of Orthopedic Surgery, Yeungnam University Medical Center, 170 Hyeonchung‑ro Nam‑gu, Daegu, 42415 Republic of Korea; 4 Department of Anesthesia and Pain Medicine, Yeungnam University Medical Center, 170 Hyeonchung‑ro Nam‑gu, Daegu, 42415 Republic of Korea

**Keywords:** Total knee arthroplasty, Revision total knee arthroplasty, New semi-constrained revision system, Septic failure, Aseptic failure, Outcome

## Abstract

**Purpose:**

The purpose of this study was to investigate the early outcomes of the new semi-constrained revision total knee arthroplasty (TKA) system by performing subgroup analysis according to the revision cause.

**Materials and methods:**

From August 2019 to July 2020, 83 revision TKAs using the fixed-bearing Attune® revision knee system with a minimum follow-up of 2 years were retrospectively reviewed. Clinically, the Knee Injury and Osteoarthritis Outcome Score for Joint Replacement, the Western Ontario and McMaster Universities Osteoarthritis Index, and range of motion (ROM) were evaluated. The incidence of systemic and specific postoperative complications was investigated. Each cohort was divided into septic (group A, 34 patients) and aseptic mode (group B, 41 patients), and compared to assess the outcomes.

**Results:**

The mean age at the time of revision was 73.3 years (range 59.0 to 84.0 years), and the follow-up duration was 36.1 months (range 30.0 to 40.0 months). Clinical outcomes and ROM significantly improved at last follow-up (*p* < 0.001). Group A showed statistically inferior clinical outcomes in the last follow-up compared to group B. Four knees (5.3%) had a postoperative femoral joint line elevation of more than 5 mm. There were no serious systemic complications. One patient underwent re-revision TKA due to recurrence of infection. No stem tip impingement or cortical erosion was observed in all patients.

**Conclusions:**

Revision TKAs using a new semi-constrained revision system showed favorable short-term follow-up outcomes, with improvement in clinical scores and ROM. Moreover, by using stem offsets, no postoperative stem tip impingement or cortical erosion was found.

**Level of evidence:**

Level IV, Retrospective Case Series.

## Introduction

Due to the greater need for primary total knee arthroplasty (TKA), the numbers of revision TKA are inevitably increasing [1]. Revision TKA can be attributed to several reasons including infection, aseptic loosening, instability, stiffness, and periprosthetic fractures [2]. Although the outcomes may vary depending on the reason for the operation, the results of revision surgery are usually poorer than that of primary TKA due to difficulties in the surgical approach, soft tissue adhesion, ligament laxity, and poor bone stock [3]. Moreover, while the outcomes of primary TKA have been systematically reported, the outcomes of revision TKA are less understood [4].

Meanwhile, although it may vary depending on the revision scenario, in general, more constrained implants are selected in revision TKA to manage ligament laxity and restore joint stability. They frequently incorporate a constrained post and cam mechanism to provide enhanced varus-valgus constraint (VVC) to supplement the function of the collateral ligaments [5]. As part of these efforts, recently, a new semi-constrained VCC revision TKA system was introduced while supplementing the predecessor (PFC Sigma TC3® knee system, DePuy Synthes, Warsaw, IN). Some recent studies reported that this new system has a more increased varus-valgus stability than the existing implant [6, 7]. Moreover, compared to the predecessor, this new system has a wider variety of weapons, such as metal augments and offset options, which may facilitate avoiding joint line elevation or stem tip impingement.

Therefore, the purpose of this study was to investigate the early outcomes of this new semi-constrained revision TKA system by performing subgroup analysis according to the revision cause. We hypothesized that favorable outcomes can be achieved in patients underwent revision TKAs using this newer revision system with this kinematic stability and a variety of options.

## Materials and methods

### Patients’ demographic characteristics

**Fig. 1:**
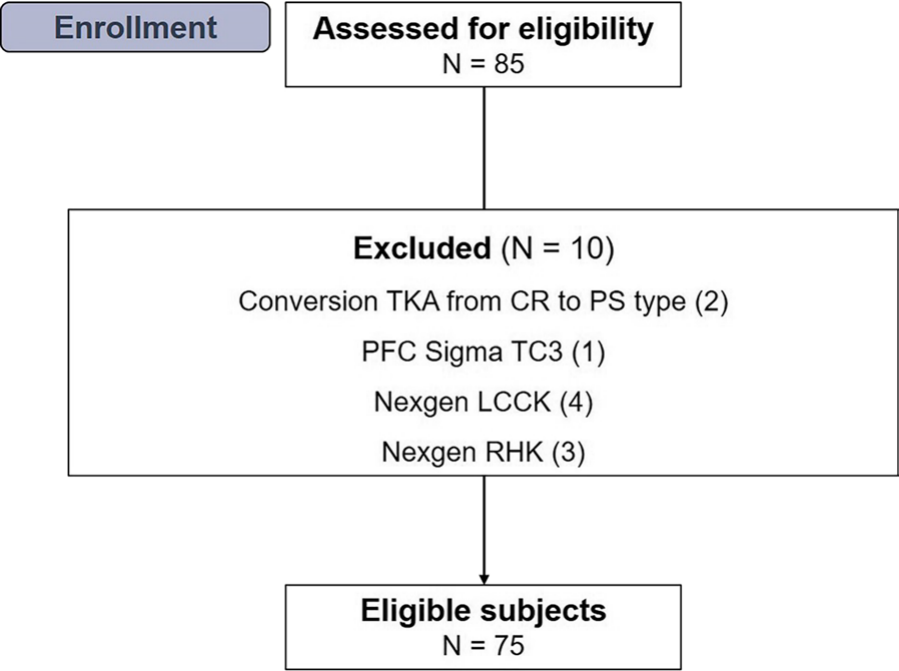
Flow diagram illustrating patient enrollment. Overall, 72 knees were enrolled in our study

This study was approved by the Institutional Review Board of our hospital, and the requirement for informed consent was waived because of its retrospective design. Between August 2019 and July 2020, we retrospectively reviewed 83 knees (78 patients) of revision TKAs replacing both femoral and tibial components. All operations were performed by a senior surgeon using the same technique at a single center. Inclusion criteria for this study were as follows: (1) revision TKA (replacement of all components) using the cemented VVC Attune® revision knee system (DePuy Synthes, Warsaw, IN, USA) with a fixed-bearing; (2) a minimum follow-up of 2 years after index operation. We excluded 8 knees with other implants during the study period: PFC Sigma TC3® used in 1 knee, legacy constrained condylar knee (NexGen® LCCK®, Zimmer Biomet Warsaw IN, USA) used in 4 knees, and rotating hinge knee prosthesis (NexGen® RHK, Zimmer Biomet, Warsaw, IN, USA) used in 3 knees; thus, 75 cases (72 patients) were enrolled in the final analysis. All cases were followed up for more than 2 years after surgery (Fig. 1).

### Subgroup analysis

We divided the modes of failure in primary TKAs into septic mode due to infection and aseptic mode due to other causes. Aseptic mode included loosening, instability, polyethylene (PE) wear, and stiffness.

**Fig. 2:**
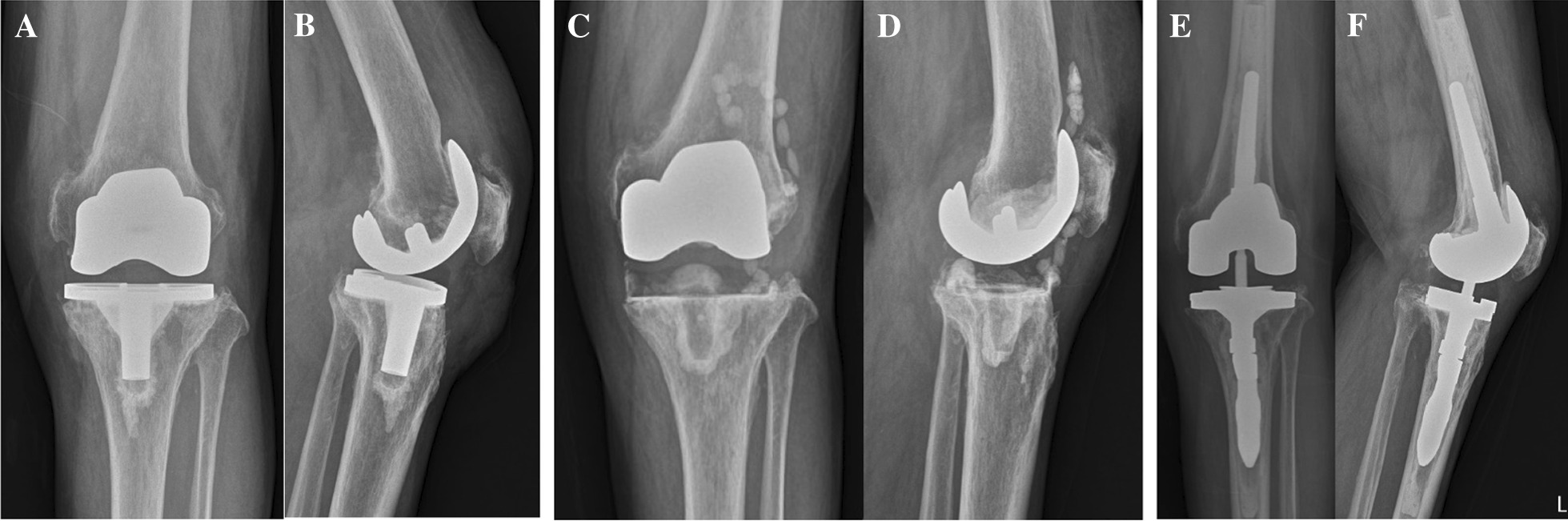
**A**, **B** About 4 years after primary TKA, an 83-year-old man was diagnosed with chronic infection of previous TKA with bony absorption in the proximal medial tibia. **C**, **D** A two-stage revision strategy was established. In the 1st stage, the autoclaved femoral component was reinserted with a rotating PE liner and antibiotics-impregnated cement. **E**, **F** Finally, a total cementation technique using susceptible antibiotics was used with cemented stems. A 2 mm offset adapter was applied for centering the tibial component. An intramedullary bone plug was inserted considering the length of the stems

All patients who underwent revision TKA for infection were diagnosed according to the latest evidence-based criteria from the International Consensus Meeting [8] and received revision surgery using a two-stage revision strategy with a minimum interval of 6 weeks. In the first operation, the implant and all cement remnants were removed. Then, a standardized radical debridement with removal of all macroscopically suspicious soft tissue and bone was performed. Finally, the autoclaved femoral component removed from the patients was reused for temporary articulating antibiotic spacer (132℃, 30 min). In addition, rotating PE liner with antibiotics-impregnated cement was inserted in the tibia that maintained the joint gap (Fig. 2C, D). Antibiotic beads were also inserted in the intramedullary canal or joint cavity, if needed. The second-stage reimplantation was planned only when there was sufficient clinical, radiographic, and laboratory evidence supporting eradication of the infection [9, 10]. The final revision was performed only when fewer than 5 polymorphonuclear leukocytes were observed in the intraoperative frozen biopsy at 400-fold magnification obtained from more than three areas and there was no gross evidence of infection during surgery.

Revision surgeries by aseptic mode were performed as a one-stage strategy [9]. Aseptic loosening was assessed by radiolucent lines (RLLs) on anteroposterior (AP) and lateral radiographs of the knee joint taken in a standardized fashion by the institutional radiology department. RLLs were defined as radiolucent intervals > 2 mm in width between either the implant and the cement or the cement and the underlying bone [11]. Zones around the TKA implants were defined as described by the Modern Knee Society Radiographic Evaluation [12].

We defined instability after TKA as abnormal and excessive displacement of the reticular elements that leads to failure of primary TKA [13]. Traumatic rupture or chronic functional attenuation of ligaments and insufficiency of extensor mechanism may be contributing factors. In the present study, among 4 cases with acute knee dislocation, three cases requiring a hinged implant due to global instability were excluded. PE wear was assessed in vivo by measuring the minimum joint space width in radiographs. Stiffness was defined as a clinical condition with limited range of motion (ROM, < 70 degrees) with or without pain after TKA [14].

In cases with multiple failure modes, two independent investigators who did not participate in surgery classified the patients to minimize any observation bias. In only two cases where consensus could not be reached, the operating surgeon re-classified them as the most fundamental cause. Some cases with PE wear were accompanied by instability, which was determined to be due to instability, the most fundamental cause [13]. Another cases, stiffness caused by septic loosening was classified into the group with septic mode.

### Surgical techniques

**Fig. 3:**
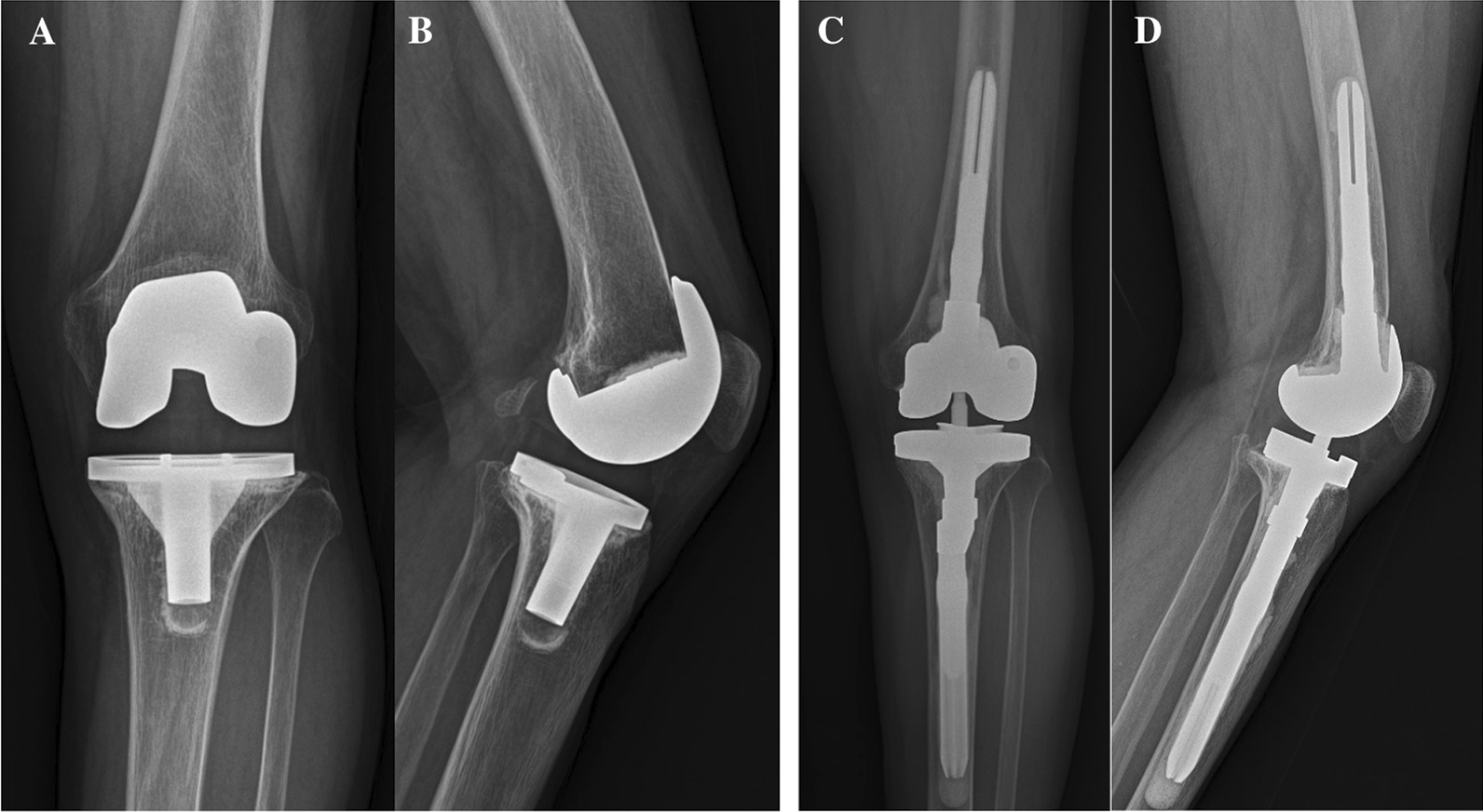
**A**, **B** Aseptic loosening of tibial component was observed on the left knee anteroposterior and lateral radiographs 10 years after primary TKA in a 77-year old woman. **C**, **D** One-stage revision TKA using a hybrid cementation technique with press-fit cementless stem was performed. Since anatomical malalignment in which the center of the tibial canal was eccentric was observed, a press-fit long stem of tibia (14 × 110 mm) with a 6 mm offset adapter was used

The rectus snip approach was performed only in 4 patients who had difficulty in joint exposure due to severe patellar baja [15]. All other revision TKAs were underwent through a medial parapatellar approach along the existing scar. The failed implants, bone cement, and debris were carefully removed being paid to minimizing bone loss. The original joint line was restored by applying distal metal augments to the femoral bone defect. We tried to confirm the accurate rotation of an appropriately sized femoral component with respect to the trans-epicondylar axis. Sequential intramedullary reaming of the femur and tibia was performed according to the planned length and thickness of the stems. Both femoral and tibial stems were used in all patients. Since this new revision system had an offset option compared to the predecessor, if it was eccentric to the canal, an offset stem was used [16]. In particular, in a revision situation where the flexion gap was large, posterior shifting of the femoral component by the posterior offset stem and additional posterior femoral augments were used to optimize the flexion gap [17]. The Anderson Orthopedic Research Institute (AORI) grade was performed intraoperatively by the operating surgeon after removal of the primary prosthesis [18]. Depending on the size and grade of the defect, autologous or allogenic structured bone grafts or trabecular metal cones were used [19]. After that, the host bone was fine-tuned and metal augmentation was applied to achieve press-fit fixation considering the level of the joint line. Finally, knee stability, patella tracking, lower limb alignment, and ROM were checked. We applied a cementing technique for all revision TKAs. In revision TKAs due to infection, a total cementation technique using susceptible antibiotics was used (Fig. 2), whereas for revision TKAs due to aseptic complications, a modified hybrid cementation technique with press-fit stem was used. The cement was applied around the implant distal to the modular junction of the stem and was also applied at the tip of both stems (Fig. 3) [17, 20].

When the total cementation technique was performed, an intramedullary bone plug was inserted considering the length of the stem. Antioxidant fixed-bearing PE inserts were used in all cases. Patellar resurfacing was not performed on all patients due to concern for infection [21].

A closed suction drain was inserted and was removed 24‒48 h after surgery. All patients applied the same perioperative pain control protocol, including multimodal drug regimen and postoperative patient-controlled analgesia. Active ROM exercise was started on the day of surgery. If normal quadriceps femoris strength was recovered on the 2nd or 3rd postoperative day, partial weight bearing with a crutch was allowed. Full weight bearing was permitted 3 weeks after surgery.

### Outcome assessments

The demographic characteristics were investigated before surgery. Clinical assessments were performed in all patients preoperatively and at last follow-up. The clinical questionnaires were assessed based on the Knee Injury and Osteoarthritis Outcome Score for Joint Replacement (KOOS-JR) [22] and the Western Ontario and McMaster Universities Osteoarthritis Index (WOMAC) for pain and function [23]. They were recorded by an independent researcher in outpatient clinic. ROM of the knee joint (including flexion contracture and further flexion angle) was measured using a long-armed goniometer by an independent physical therapist. The values at the final follow-up were compared with the preoperative values. For subgroup analysis, patients were divided into group A (septic mode) and B (aseptic mode).

**Fig. 4:**
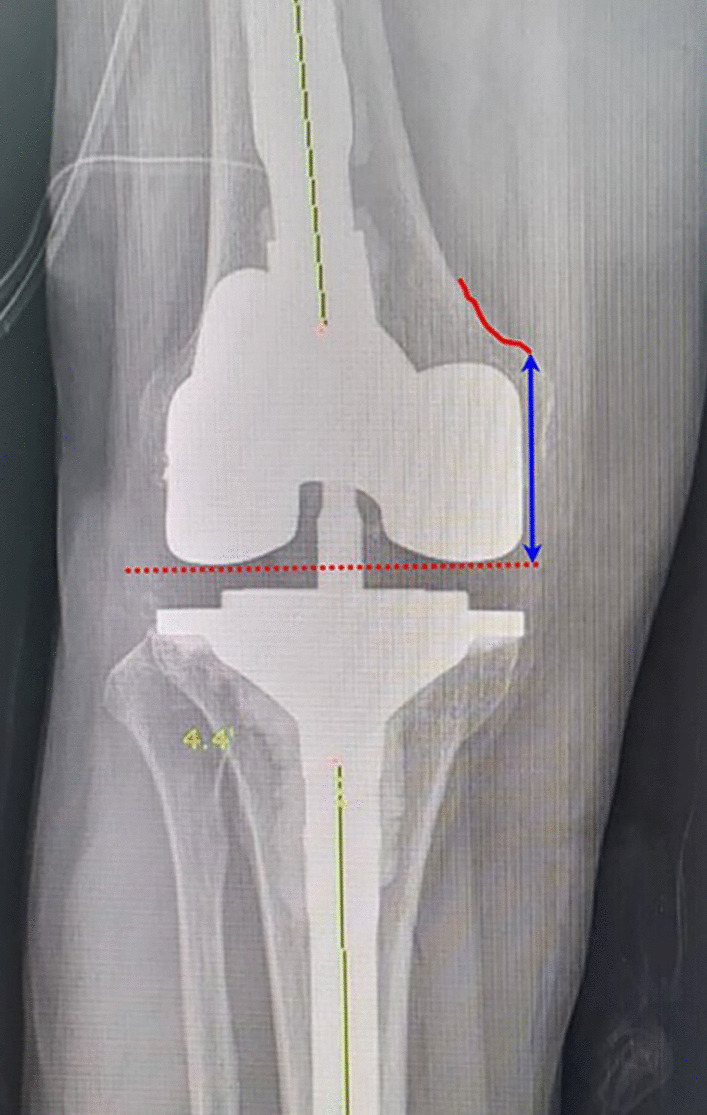
Femoral joint line position was defined as the distance from the adductor tubercle to the joint line in an anteroposterior radiograph (blue-colored arrow)

Bilateral standing AP and lateral radiography of the knee joint, Merchant view, and lower-extremity scanography were performed preoperatively; at 3, 6, 12, and 24 months postoperatively; and then every year until the last follow-up. All radiographic measurements were digitally acquired using a picture archiving and communication system (Maroview®, version 5.4; Marotech, Seoul, Korea) in the format of DICOM (Digital Imaging and Communicating in Medicine). Radiographic outcomes included the hip–knee–ankle (HKA) angle (with varus alignment as a negative value) and posterior tibial slope angle (PTSA, the angle between the tangent to the medial tibial plateau and the perpendicular line to the proximal tibial anatomic axis) [24]. The positions of femoral and tibial components were measured using the α, β, γ, and δ angles according to the Knee Society Radiographic Evaluation method [25]. Moreover, RLLs were investigated through AP and lateral radiographs [11]. Changes of femoral joint line after index operation were assessed. The femoral joint line position was defined as the distance from the adductor tubercle to the joint line in an AP radiography (Fig. 4) [26].

The incidence of postoperative complications was documented via chart review. In addition to surgery-related complications, systemic complications were also investigated. Systemic complications were defined as exacerbation of underlying systemic disease or development of a new medical problem [25].

### Statistical analysis

Statistical evaluation was performed using IBM SPSS software (Version 28; IBM Co., Chicago, IL, USA), and continuous data were expressed as means with SDs. All dependent variables were tested for normality of distribution and equality of variances using the Kolmogorov–Smirnov test and analyzed using parametric or nonparametric tests based on normality. According to normality test, the paired *t* test was used to compare the preoperative and postoperative clinical and radiographic outcomes. Intergroup comparisons were made using independent sample *t* test. The Fisher exact test was used to compare ratios between the groups. For all tests, *p* < 0.05 was considered statistically significant.

## Results

Patient demographic characteristics and operative details are summarized in Tables 1 and 2, respectively. The mean age at the time of revision surgery was 73.3 years (range 59.0 to 84.0 years). The mean interval from primary TKA to revision TKA was 6.7 years (range 0.8 to 15 years), and the follow-up duration was 36.1 months (range 30.0 to 40.0 months). There were more modes of failure by aseptic complications (group B, 41 patients, 54.7%), and among them, aseptic loosening was the most common.

**Table 1:** Patient demographic characteristics

Variable	Value (total = 75)
Age, years*	73.3 (59.0‒84.0)
Sex, *n*^†^	
Female, *n*	58 (77.3)
Male, *n*	17 (22.7)
BMI, kg/m^2*^	26.9 (20.2‒31.2)
ASA class	
1	3 (4.0)
2	45 (60.0)
3	26 (34.7)
4	1 (1.3)
F/U period, months*	36.1 (30.0‒40.0)
Side, *n*^†^	
Right, *n*	34 (45.3)
Left, *n*	41 (54.7)
Time interval from primary TKA to revision TKA, years^*^	6.7 (0.8‒17.0)
Modes of failure, *n*^†^	
Septic	34 (45.3)
Aseptic	41 (54.7)
Loosening	21 (28.0)
Instability	11 (14.7)
PE wear	7 (9.3)
Stiffness	2 (2.7)

BMI, body mass index; ASA, American Society of Anesthesiology Classification System; F/U, follow-up; TKA, total knee arthroplasty; and PE, polyethylene

*Data are presented as mean (range)

^†^Data are presented as number (percentage)

**Table 2:** Operative details

Variable	No. (total = 75)
Operation time, min*	118.5 (68.0‒149.0)
Polyethylene thickness, mm*	12.7 (8.0‒16.0)
Need for blood transfusion, *n*^†^	9 (12.0)
Cementation technique, *n*^†^	75 (100)
Total cementation with fully cemented stem	34 (45.3)
Hybrid cementation with press-fit stem	41 (54.7)
Stemmed implant (both femur and tibia), *n*^†^	75 (100)
Metal augments, *n*^†^	75 (100)
Femur (distal)	75 (100)
Femur (posterior)	63 (85.3)
Tibia (proximal)	68 (90.7)
Bone graft, *n*^†^	50 (66.7)
Allogenic structured graft	48 (64)
Trabecular metal cone	2 (2.7)

Blood transfusions were performed when hemoglobin < 8 g/dL

*Data are presented as mean (range)

^†^Data are presented as number (percentage)

In subgroup analysis, only hospital stay and 2-stage revision showed significant differences between the groups (Table 3).

**Table 3:** Comparison of demographic, operative, and radiologic parameters between the groups

	Group A (septic)	Group B (aseptic)	*p *value
Knees, *n* (%)^*^	34 (45.3)	41 (54.7)	–
Age, years^†^	69.3 (59.0‒82.0)	73.3 (67.0‒84.0)	0.105
Sex, M/F^*^	6/28	11/30	0.344
BMI, kg/m^2†^	26.8 (20.2‒30.8)	26.9 (23.2‒31.2)	0.613
ASA class 3/4^*^	15/34 (44.1)	12/41 (29.3)	0.182
Hospital stay, day^†^	18.5 (14.0‒28.0)	12.9 (7.0‒21.0)	0.035
F/U period, months^†^	36.3 (30.0‒39.0)	35.9 (29.0‒40.0)	0.512
Time interval from TKA to revision, years^†^	4.7 (0.8‒8.0)	8.3 (1.5‒17.0)	0.031
2nd-stage revision, *n* (%)^*^	34 (100.0)	–	< 0.001
Operation time, min^†^	115. 9 (68.0‒138.0)	121.5 (85.0‒149.0)	0.107
PE thickness, mm^†^	12.8 (8.0‒14.0)	12.5 (8.0‒16.0)	0.621
Pre-revision HKA angle, °^‡^	− 5.9 ± 4.1	− 5.6 ± 3.9	0.560
Post-revision HKA angle, °^‡^	− 2.1 ± 3.3	− 2.4 ± 3.7	0.379
Pre-revision PTSA, °^‡^	3.3 ± 1.8	3.1 ± 1.9	0.780
Post-revision PTSA, °^‡^	5.5 ± 2.6	5.2 ± 2.2	0.603
Component position, °^‡^			
* α* angle	94.5 ± 3.8	94.9 ± 3.3	0.439
*β* angle	90.5 ± 2.7	89.8 ± 2.4	0.713
*γ* angle	3.2 ± 4.0	3.6 ± 4.3	0.527
*δ* angle	87.5 ± 3.8	87.0 ± 3.7	0.626
Femoral JL position, mm^‡^			
Pre-revision	41.0 ± 7.2	40.7 ± 6.3	0.481
Post-revision	42.2 ± 6.6	42.9 ± 6.1	0.632
≥ 5 mm of femoral JL elevation, *n* (%)^*^	1 (2.9)	3 (7.3)	0.401

BMI, body mass index; ASA, American Society of Anesthesiology Classification System; F/U, follow-up; PE, polyethylene; HKA, hip–knee–ankle; PTSA, posterior tibial slope angle; and JL, joint line

^*^Data are presented as numbers

^†^Data are presented as means ± range

^‡^Data are presented as means ± standard deviation

A negative value of HKA angle indicated varus alignment

Operation time indicated the time during index revision surgery

^†^Independent sample *t* test was used for intergroup comparisons (*p* < 0.05)

^*^The Fisher exact test was used to compare ratios between the groups

All clinical evaluations and knee joint ROM significantly improved at last follow-up (*p* < 0.001). Group A by septic mode showed statistically inferior clinical outcomes in the last follow-up compared to group B by aseptic mode (Table 4).

**Table 4:** Comparison of clinical outcomes during the follow-up period

	Total	Group A (septic)	Group B (Aseptic)	*p* value^†^
(A) KOOS-JR scores				
Pre-op	38.8 ± 12.5	38.3 ± 12.2	39.1 ± 12.7	0.738
PO at 3 months	42.1 ± 6.5	41.9 ± 6.0	44.3 ± 6.7	0.021
PO at 6 months	42.1 ± 6.5	41.9 ± 6.0	44.3 ± 6.7	0.021
PO at 12 months	50.2 ± 4.3	48.3 ± 3.2	55.2 ± 5.5	< 0.001
PO at 24 months	53.3 ± 3.3	48.7 ± 3.5	55.8 ± 3.1	< 0.001
*p* value^‡^	< 0.001	< 0.001	< 0.001	
(B) WOMAC (pain and function)				
Pre-op	60.8 ± 7.6	60.2 ± 8.1	61.8 ± 7.2	0.461
PO at 3 months	42.9 ± 4.6	47.2 ± 4.1	39.8 ± 5.2	< 0.001
PO at 6 months	42.9 ± 4.6	47.2 ± 4.1	39.8 ± 5.2	< 0.001
PO at 12 months	40.5 ± 4.3	45.6 ± 4.1	34.1 ± 4.4	< 0.001
PO at 24 months	39.8 ± 4.1	44.6 ± 4.0	33.1 ± 4.1	< 0.001
*p* value^‡^	< 0.001	< 0.001	< 0.001	
(C) ROM of the knee joint				
FC (°)				
Pre-op	9.4 ± 6.8	10.3 ± 9.3	8.5 ± 5.8	0.039
PO at 3 months	4.5 ± 7.8	6.0 ± 7.3	3.5 ± 8.8	0.015
PO at 6 months	4.5 ± 7.8	6.0 ± 7.3	3.5 ± 8.8	0.015
PO at 12 months	4.3 ± 6.8	5.7 ± 6.5	2.1 ± 7.0	< 0.001
PO at 24 months	4.3 ± 6.2	5.6 ± 6.4	2.2 ± 6.1	< 0.001
*p* value^‡^	< 0.001	< 0.001	< 0.001	
FF (°)				
Pre-op	92.3 ± 7.9	78.4 ± 8.1	105.3 ± 7.4	0.027
PO at 3 months	110.3 ± 8.9	108.6 ± 8.8	115.4 ± 9.2	0.01
PO at 6 months	110.3 ± 8.9	108.6 ± 8.8	115.4 ± 9.2	0.01
PO at 12 months	116.1 ± 7.3	110.4 ± 7.1	123.0 ± 7.4	< 0.001
PO at 24 months	117.4 ± 9.3	111.7 ± 9.1	122.8 ± 9.8	< 0.001
*p* value^‡^	< 0.001	< 0.001	< 0.001	

(A) KOOS-JR, Knee Injury and Osteoarthritis Outcome Score for Joint Replacement; Pre-op, preoperative; and PO, postoperative

(B) WOMAC, Western Ontario and McMaster Universities Osteoarthritis Index; Pre-op, preoperative; and PO, postoperative

(C) ROM, range of motion; FC, flexion contracture; F/U, follow-up; FF, further flexion; Pre-op, preoperative; and PO, postoperative

Data are presented as mean ± standard deviation

^†^Group B by aseptic mode showed superior outcomes (independent sample *t* test, *p* < 0.05)

^‡^Clinical outcomes were improved after the index operation (paired *t* test, *p* < 0.05)

Pulmonary thromboembolism did not occur, and proximal deep vein thrombosis was found in 3 patients (4.0%). A new oral anticoagulant, apixaban, was administered. Although there was no serious hepatic failure, mild elevation of hepatic enzyme level was observed in 8 patients (10.7%). After the operation, dysuria was observed in 10 (13.3%) patients, but most of them improved after administration. One patient underwent re-revision TKA including debridement and thicker PE exchange due to recurrence of infection. No stem tip impingement or cortical erosion was observed in all patients (Table 5).

**Table 5:** The incidence of systemic and specific complications

		Total	Group A (Septic)	Group B (Aseptic)	*p* value^†^
	Cardiovascular	2 (2.7)	1 (1.3)	1 (1.3)	–
	Pulmonary	3 (4.0)	2 (2.7)	1 (1.3)	–
	Gastrointestinal	–	–	–	–
	Hepatic	8 (10.7)	5 (6.7)	3 (4.0)	
*Systemic*	Nephrotic	–	–	–	–
	Endocrinologic	–	–	–	–
	Urologic	10 (13.3)	6 (8.0)	4 (5.3)	–
	Cerebral	1 (1.3)	–	1 (1.3)	–
	Delirium	8 (10.7)	3 (4.0)	5 (6.7)	
	Venous thromboembolism				
	*PTE*	–	–	–	–
	*DVT (proximal)*	3 (4.0)	2 (2.7)	1 (1.3)	
*Specific*	*DVT (distal)*	8 (10.7)	4 (5.3)	4 (5.3)	
	Stem tip impingement, cortical erosion	–	–	–	–
	Infection	1 (1.3)	1 (1.3)	–	
	Periprosthetic fracture	–	–	–	–

PTE, pulmonary thromboembolism; DVT, deep vein thrombosis

Data are presented as number (percentage)

## Discussion

This study informs a comprehensive and descriptive review of outcomes after revision TKA using a newly developed semi-constrained revision system in a relatively large number of patients with a mean follow-up of 2 years. To our knowledge, although some biomechanical cadaveric studies of this new design of revision system have been reported [6, 7], clinical studies on outcomes are still lacking. The outcomes following revision TKA performed using this new prosthesis are first data in the published literature. All patients in this cohort underwent the fixed-bearing Attune® revision knee system in revision TKA performed by an experienced arthroplasty surgeon at a single institution. The perioperative variables in the current study can be used to guide understanding of the factors that affect patient outcomes after revision TKA performed using this new prosthesis.

In scenarios with severe distal femoral bone loss of revision TKA, the geometry of the femoral condyles is sometimes not restored, resulting in elevation of the femoral joint line [27]. Accordingly, when the femoral joint line was preserved after revision TKA, clinical outcomes, especially postoperative ROM, could be guaranteed [26]. This may lead to the fact that distal femoral augments may be used more frequently in revision TKA. As with these implants, the more options available for distal femoral augments such as 8 mm or 12 mm, the more physiological or native anatomical joint lines can be preserved. In the current study, only 4 knees (5.3%) of patients showed a femoral joint line elevation more than 5 mm, which resulted in a relatively well-preserved femoral joint line.

The postoperative clinical outcomes were poorer in the group with septic mode than in the group with aseptic mode (Table 4). A study reported outcomes after revision TKAs in 125 patients with a mean follow-up of 36 months, and septic revision cases showed inferior function and clinical outcomes after surgery [28]. In another study that reported 2-year outcomes of 150 revision TKAs, it was reported that the reason for revision TKA was predictive of outcome. They reported that revision TKA due to aseptic loosening showed better results for satisfaction, functional improvement, and complication rates [14]. As with the previous studies [4, 28], the present study showed that group with septic mode revealed statistically inferior clinical outcomes after revision TKAs. As shown in Table 3, in revision TKA by septic mode, all patients (34 knees in 34 patients) underwent 2nd-stage strategies (Table 3). This may be due to the fact that the ROM of the knee joint was not sufficient during the interval of at least 6 weeks after the 1st-stage antibiotics-impregnated cement insertion, and the adhesion of the surrounding soft tissues was severe due to infection.

Meanwhile, no stem tip impingement or cortical erosion was observed in the present study. Stem offsets are often required when there is an anatomical malalignment between the center of the diaphyseal canal of the tibia and the center of the tibial plateau (Figs. 2E, 3C) [17]. Moreover, offset reduces bone-to-implant stress by allowing stability in flexion, ligament balancing, and optimal bone coverage [29]. Offset option was not available on PFC Sigma TC3®, which could be a significant limitation when using cementless stems [17]. Accordingly, overhang of the tibial tray, stem tip impingement or cortical erosion could be induced. In a study on the results of PFC Sigma TC3, it was reported that end-of-stem pain was found in 8 out of 31 patients (25.8%) [30]. Since this new revision system had an offset option compared to the predecessor, if it was eccentric to the canal, an offset stem was used [16]. No stem tip impingement or cortical erosion was observed in all patients.

Despite the informative results of this study, it has some limitations that need to be considered. First, the relatively short follow-up period may be a major concern. Although this was a short-term follow-up study, revision TKA using this prosthesis showed relatively favorable outcomes. All clinical outcomes improved after index operation. The duration of follow-up (minimum, 24 months; mean, 27.2 months) represents short-term outcomes; accordingly, survival and satisfaction may not be maintained from this time point forward. Future follow-up is required to assess the change in outcomes over time within this cohort. However, when compared with other studies in terms of clinical outcomes after revision TKAs [4, 31], the outcomes of the current study were favorable. Moreover, as reported in studies of patterns of functional improvement after revision TKA, we consider the change in ROM after one year postoperatively to be minimal in most patients [32]. Therefore, although this was a follow-up study of at least 2.5 years, it was meaningful that the degree of improvement in ROM in the present study was comparable to that of previous studies [4, 25]. Nevertheless, since significant differences may have been missed, mid- to long-term studies with this new implant are needed. Second, since this study was not a comparative study with a group that implemented other types of implants, it was difficult to guarantee that this new system showed better outcomes than the predecessors. Therefore, a long-term comparative study is needed to confirm that this new revision system can provide better outcomes compared to other implants. Finally, a female predominance was observed in the present study. Thus, the outcomes may not be the same for populations with different sex ratios. However, osteoarthritis is known to be more prevalent in women in Asia. In particular, the rate of primary TKA in Korean women is about 5 to 7 times higher than that of men.

## Conclusion

Revision TKAs using a new semi-constrained revision system showed favorable short-term follow-up outcomes, with improvement in clinical scores and ROM. Moreover, by using stem offsets, no postoperative stem tip impingement or cortical erosion was found.

## Data Availability

Not applicable.
